# Genome-wide characterization and expression analysis of MYB transcription factors in *Chrysanthemum nankingense*

**DOI:** 10.1186/s12870-023-04137-7

**Published:** 2023-03-14

**Authors:** Penghui Ai, Jundong Xue, Zhongya Shi, Yuru Liu, Zhongai Li, Tong Li, Wenqian Zhao, Muhammad Ayoub Khan, Dongru Kang, Kangxiang Wang, Zicheng Wang

**Affiliations:** 1 State Key Laboratory of Crop Stress Adaptation and Improvement, Plant Germplasm Resources and Genetic Laboratory, Kaifeng Key Laboratory of Chrysanthemum Biology, School of Life Sciences, Henan University, Jinming Road, Kaifeng, 475004 Henan China; 2 Technology&Media University of Henan Kaifeng, Jinming Road, Kaifeng, 475004 Henan China

**Keywords:** CnMYB, Chrysanthemum, Drought stress, Salt stress, CpG island

## Abstract

**Background:**

Chrysanthemum is a popular ornamental plant worldwide. MYB (v-myb avian myeloblastosis viral oncogene homolog) transcription factors play an important role in everything from stress resistance to plant growth and development. However, the MYB family of chrysanthemums has not been the subject of a detailed bioinformatics and expression investigation.

**Results:**

In this study, we examined 324 CnMYB transcription factors from *Chrysanthemum nankingense* genome data, which contained 122 Cn1R-MYB, 183 CnR2R3-MYB, 12 Cn3R-MYB, 2 Cn4R-MYB, and 5 atypical CnMYB. The protein motifs and classification of CnMYB transcription factors were analyzed. Among them, motifs 1, 2, 3, and 4 were found to encode the MYB DNA-binding domain in R2R3-MYB proteins, while in other-MYB proteins, the motifs 1, 2, 3, 4, 5, 6, 7, and 8 encode the MYB DNA-binding domain. Among all *CnMYBs*, 44 genes were selected due to the presence of CpG islands, while methylation is detected in three genes, including *CnMYB9*, *CnMYB152*, and *CnMYB219*. We analyzed the expression levels of each *CnMYB* gene in ray floret, disc floret, flower bud, leaf, stem, and root tissues. Based on phylogenetic analysis and gene expression analysis, three genes appeared likely to control cellulose and lignin synthesis in stem tissue, and 16 genes appeared likely to regulate flowering time, anther, pollen development, and flower color. Fifty-one candidate genes that may be involved in stress response were identified through phylogenetic, stress-responseve motif of promoter, and qRT-PCR analyses. According to genes expression levels under stress conditions, six *CnMYB* genes (*CnMYB9*, *CnMYB172*, *CnMYB186*, *CnMYB199*, *CnMYB219*, and *CnMYB152*) were identified as key stress-responsive genes.

**Conclusions:**

This research provides useful information for further functional analysis of the *CnMYB* gene family in chrysanthemums, as well as offers candidate genes for further study of cellulose and lignin synthesis, flowering traits, salt and drought stress mechanism.

**Supplementary Information:**

The online version contains supplementary material available at 10.1186/s12870-023-04137-7.

## Background

Transcription factors (TFs) bind to the DNA regulator sequence to regulate plant growth and development. They can also boost plant tolerance to abiotic and biotic stressors by orchestrating regulatory networks. The MYB family is a prominent TF family in eukaryotes that regulates a variety of biological processes [1]. The name of MYB TFs derives from their conserved N-terminal MYB DNA-binding domain (DBD) repeats (Rs), which usually contain 52 amino acid residues per repetition. The MYB domain comprises one to four imperfect amino acid repeats. Accordingly, the MYB protein family can be divided into four subclasses based on the number of MYB domains: 1R, R2R3-, 3R-, and 4R-MYB proteins [1].

The first MYB gene was discovered in maize in 1997 [2]. Since then, a considerably wider variety of plant species, such as *Arabidopsis thaliana* [3], *Oryza sativa* (rice) [4], *Morella Rubra* (Chinese bayberry) [5], *Cajanus Cajan* (pigeon pea) [6], etc. have shown evidence of the MYB family in genome-wide analyses. Previous studies have shown that the MYB family participates in diverse processes, including primary and secondary metabolism [1, 7–11], root hair development [12–14], flower development [15, 16], and responses to biotic and abiotic stresses [1, 17–27].

DNA methylation is a conserved epigenetic mark that is important for the development and stress responses in plants and many animals [28]. The CpG islands in plant genomes are more frequently methylated, and these variations in methylation status at promoters limit or increase the capacity of transcription factor (TF) proteins to bind to the DNA promoter, which in turn inhibits or increases the transcriptional activity of genes [28, 29]. Although multiple studies suggested that DNA methylation controlled anthocyanin accumulation and the MYB TFs’ role in the stress response [30, 31]. Studies on the effect of methylation in controlling MYB TF activities via CpG islands are scarce.

The productivity and quality of the commercially grown ornamental plant *Chrysanthemum morifolium Ramat* are considerably impacted by harsh environmental conditions [32]. The MYB TF family, which are essential transcription factors in the plant stress response system, should be crucial for the growth and development of chrysanthemums. *Chrysanthemum*, on the other hand, has a substantial number of hybrid polyploidy variants lacking high-quality reference genomes. Because there is currently a dearth of reliable information about the *Chrysanthemum* reference genome, only a portion of the MYB family of genes has been studied in *Chrysanthemum* [33]. It has become crucial to conduct genome-wide analysis of the MYB superfamily in chrysanthemum. Fortunately, a whole-genome fine mapping of *C. nankingense* was created using next-generation sequencing [34]. *C. nankingense* is one of the progenitors of *Chrysanthemum×morifolium* (Ramat) [32]. Due to its simple diploid genetic background, it is suitable to use as a model plant in genetic studies. Identification and incorporation of important genes into commercial varieties improve the quality and stress tolerance of the plant. Therefore, it is of great significance to identify the *MYB* family genes in *C. nankingense*.

In this study, 324 *MYB* genes were found in the genome of *C. nankingense* (http://www.amwayabrc.com). In addition, we looked into the MYB family genes’ evolutionary relationships, conserved protein domains, exon-intron architectures, cis-acting elements, and recognition of CpG islands. We further analyzed the expression patterns of these genes in different tissues based on RNA-Seq data. Finally, a phylogenetic tree that included 51 CnMYBs and 64 functional MYBs regulating flavonoid biosynthesis or defense responses from other species was generated in order to find additional CnMYBs involved in the regulation of flavonoid biosynthesis and defense responses in *C. nankingense*. The expression levels of selected 18 response genes and the methylation status of 3 genes of the *CnMYB* genes family in shoots under salt and drought stresses were then analyzed by quantitative real-time PCR and methylation-specific polymerase chain reaction (MSP). Together, these findings lay a strong framework for further research into the functions of the CnMYB gene family in *C. nankingense* under various environmental stresses.

## Results

### Identification and sequence features of *MYB* genes in *Chrysanthemum nankingense*

**Fig. 1:**
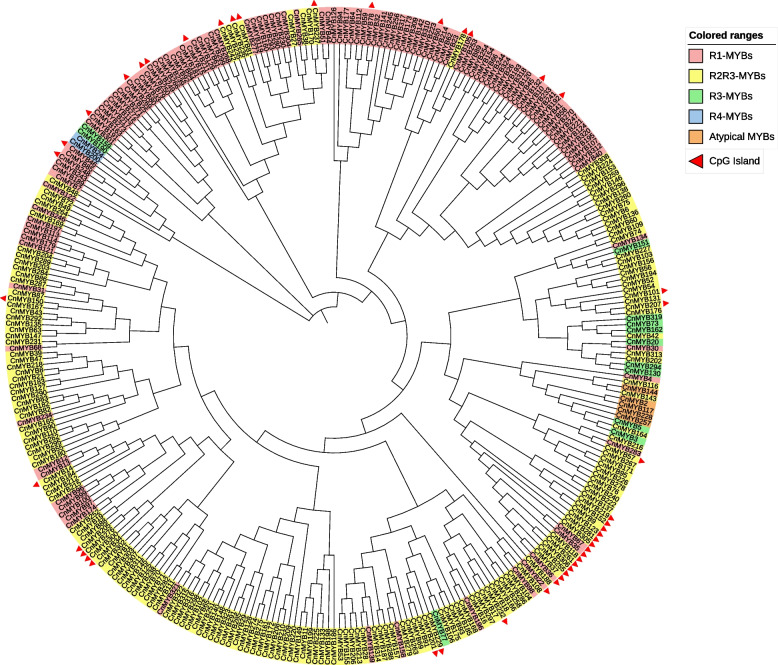
A phylogenetic tree of 324 CnMYB proteins of *Chrysanthemum nankingense*. The tree was constructed in MEGA 7.0. The classes are shown in different colors and 44 *CnMYB* genes containing CpG islands are marked with a red triangle

To identify *CnMYB* genes present in the *C. nankingense* genome, the MYB domain (PF00249) from the Pfam database was used as a query in the HMM search against the genome. A total of 324 deduced amino acid sequences that might contain MYB repeats or MYB-like repeats were obtained, named CnMYB1 to CnMYB324. All putative MYBs were further examined by the NCBI conserved Domain Search and SMART program for the presence of the MYB DNA-binding domains. Four different subfamilies were differentiated based on the number and location of domain repeats, including 1R-MYB (117), R2R3-MYB (183), 3R-MYB (12), and 4R-MYB (2) genes (Fig. 1, Table S1). A phylogenetic tree of CnMYBs was constructed by aligning the whole set of MYB protein sequences from *C. nankingense* (Fig. 1). Based on the genome data, five CnMYB contained five or six complete MYB domains, and therefore these five MYB members are classified as atypical MYBs (Table S1). These genes’ complete information includes gene name, gene ID, protein length, molecular weight (MW), isoelectric point (pI), and scaffold location is available in Table S1. The protein length varied, ranging from 70 amino acids to 1451 amino acids, the MW ranged from 8.03 to 162.55 kDa, and the pI varied from 4.08 to 11.51. Since the genome information of *C.nankingense* did not provide chromosome information, all 324 *CnMYB* genes were located in 289 scaffolds (Table S1).

CpG islands, which are rich in CG, are generally located near the promoters of genes [28, 29]. DNA methylation is higher in these regions in plant genomes [29]. CpG islands were detected in 44 members (Fig. 1, Table S2). Amongst them, ten genes contained CpG islands in the promoter region, 19 members were found to have it in the gene body, and 18 in the cross-promoter and gene region. Three members have two CpG islands, and the island length ranged from 305 to 965 bp (Table S2).

### The classification, motif composition, and gene structure of the *CnMYB* gene family in *C. nankingense*

**Fig. 2:**
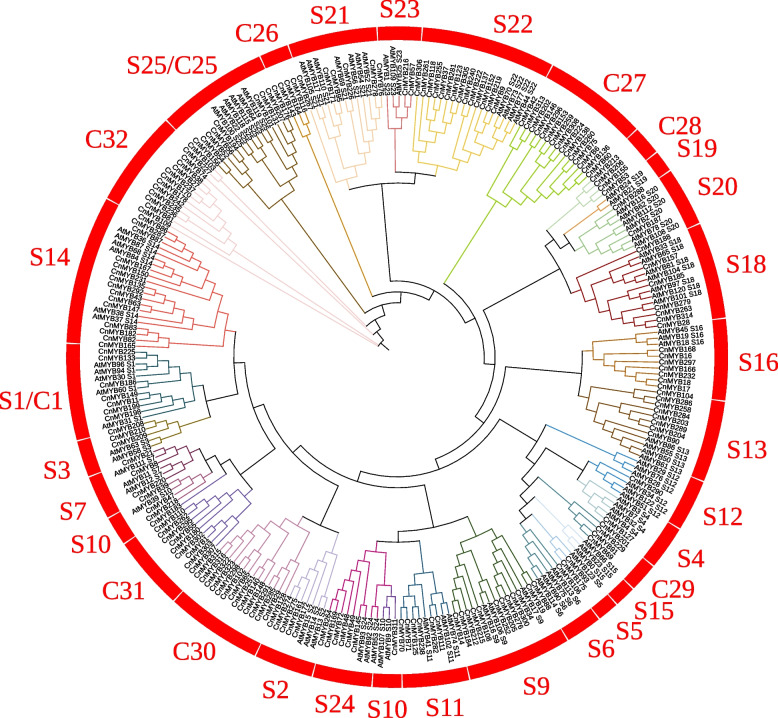
Phylogenetic relationships of R2R3-MYB proteins between *Chrysanthemum nankingense* and *Arabidopsis*. The tree was constructed in MEGA 7.0. All 32 subfamilies of R2R3-MYBs were well separated in different clades and represented by different colors

To further classify and understand the functions of the *R2R3-CnMYB* gene family members, phylogenetic analyses were carried out based on complete protein sequences of 183 R2R3-CnMYBs and 126 R2R3-AtMYBs. Based on the R2R3-AtMYBs classification principle [1], 183 R2R3-CnMYBs can be divided into 32 subfamilies (C1-C32) (Fig. 2). The C1-C25 subfamilies corresponded to S1-S25 in *Arabidopsis*. C1-C25 subfamilies included 117 R2R3-CnMYBs. There were seven specific clades (C26-C32) in *C. nankingense* that did not cluster with *Arabidopsis*, and none of the R2R3-CnMYBs belonged to the S6, S15, S19, and S23 *Arabidopsis* subfamilies. The largest clade was C27 with 15 R2R3-CnMYBs. Other CnMYBs (1R-MYB, 3R-MYB, 4R-MYB, and atypical MYBs) proteins could be divided into I-XVI subfamilies (Fig. S2).

**Fig. 3:**
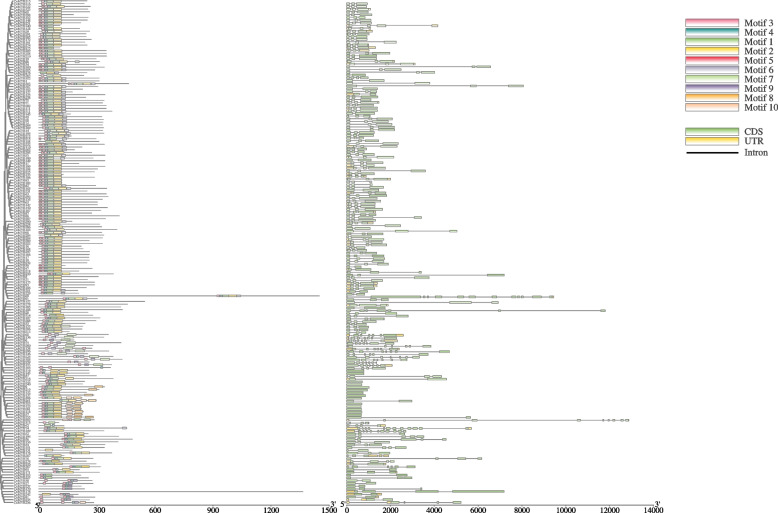
Conserved motif and gene structure analysis of *Chrysanthemum nankingense R2R3-MYB* genes. **A** Motif distribution of *R2R3-MYB* genes. **B** Gene structure of *R2R3-MYB* genes. The tree was constructed in MEGA 7.0 using the full-length amino acid sequences of 183 R2R3-MYBs in *Chrysanthemum nankingense*. The ten conserved motifs are shown in different colors and their specific sequence information is provided in Supplementary Fig. S1. The exon-intron structural diagram shows CDS, UTR, and introns as green boxes, yellow boxes, and black lines, respectively

Ten conserved motifs were identified in the R2R3-CnMYBs proteins by MEME software, motif 1, motif 2, motif 3, and motif 4 were found to encode the MYB DNA-binding domain, while the other motifs didn’t have function annotation (Fig. 3A, Fig. S1). Each R2R3-CnMYB protein contained one to seven conserved motifs. Most R2R3-CnMYB proteins (83%) contain a motif group that has 1, 2, 3, and 4 motifs, with C26 (CnMYB116, CnMYB164, and CnMYB143), C27 (CnMYB60, CnMYB163, et al.,) and C32 (CnMYB103, CnMYB227, et al.,) subfamilies lacking this motif group (Fig. 3A). Other CnMYB proteins (1R-MYB, 3R-MYB, 4R-MYB, and atypical MYBs) have one to ten conserved motifs (Fig. S3). Most proteins contain two motifs, VIII, X, XII, XIV, and XVI had several motifs (Fig. S2A). The MYB DNA-binding domains were represented by motifs 1, 2, 3, 4, 5, 6, 7, and 8 (Fig. S3).

As shown in Fig. 3B, the structure of R2R3-CnMYBs was also examined. The 183 *R2R3-MYB* genes contained different number of exons, varying from 1 to 13, with an average of 3.2. Most of the *R2R3-MYB* genes had three (109/183) exons and accounted for approximately 60% of *R2R3-MYB* gene family members, whereas 23% and of *R2R3-MYB* genes have less than three exons, while 18% have more than three exons. In addition, most of the *R2R3-MYB* genes clustered in C26 (CnMYB109, CnMYB236 et al.,) and C28 (CnMYB42, CnMYB313, et al.,) having more than three exons. As shown in Fig. S2B, the number of exons in other *CnMYB* genes (*1R-MYB, 3R-MYB, 4R-MYB,* and *atypical MYBs*) ranged from one to eighteen. In addition, combined with phylogenetic analysis, most genes clustered in the same group had the same or a similar number of exons (Fig. S2B).

### Expression profiles of *CnMYB* genes in different tissues

**Fig. 4:**
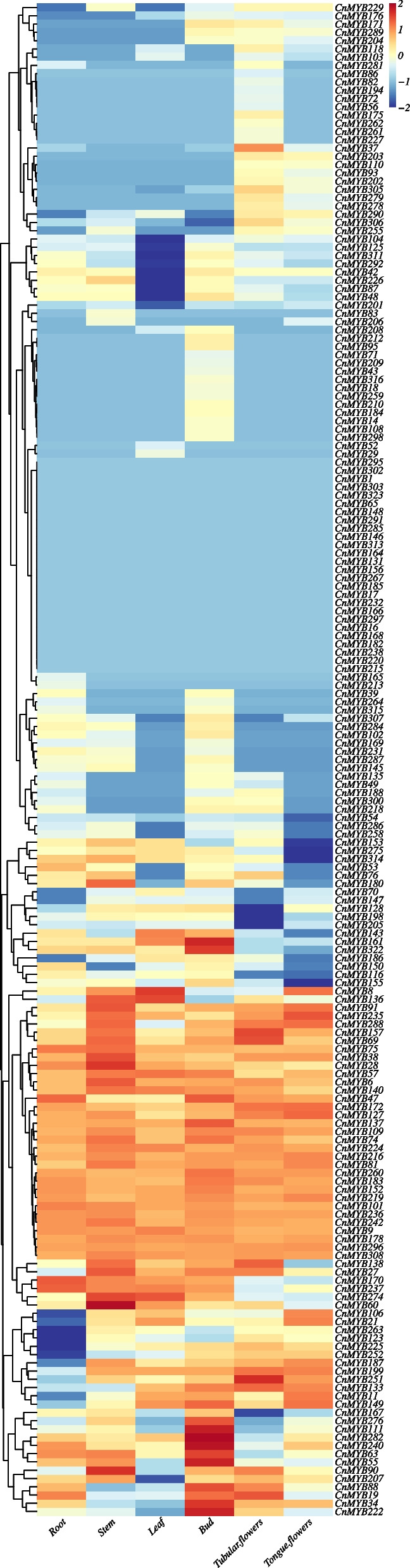
Heatmap and clustering diagram showing the expression patterns of chrysanthemum *R2R3-MYBs* genes in different tissues. The color scale represents log2 expression values, blue represents low expression and red indicates a high expression level

The expression trends of *CnMYB* genes in different tissues, such as root, stem, leaf, flower bud, tubular flower, and tongue flower, were calculated by using FPKM values based on previously generated Illumina RNA-Seq data [34]. An expression heatmap of the *CnMYB* genes was generated (Fig. 4, Fig. S4). The *R2R3-MYB* genes expression results showed that 64 genes (FPKM> 0) had expression and 26 genes (FPKM = 0) were not expressed in any tissue (Table S3). The number of *R2R3-MYB* genes expressed in the root, stem, leaf, bud, tubular flowers, and tongue flowers was 62, 73, 61, 93, 72, and 55, respectively (Table S3), the expression level of these genes was over 1 (FPKM) (Table S3). *R2R3-MYB* genes expression were highest in the flower bud tissue and lowest in the tongue flowers (Fig. 4, Table S3).

Based on transcriptome data, 85 *R2R3-MYB* genes (FPKM> 0) were expressed in all three flower tissues. Among them, *CnMYB219*, *CnMYB152*, *CnMYB242*, *CnMYB308*, *CnMYB81*, *CnMYB224*, *CnMYB127*, and *CnMYB296* genes were highly expressed in the three tissues (FPKM> 20) (Table S3). We also identified genes that were specifically expressed in the bud (23 genes, *CnMYB167*, *CnMYB102*, *CnMYB212*, *CnMYB95*, and *CnMYB284* genes were highly expressed in this tissue (FPKM> 3)), tubular flower (10 genes, *CnMYB175*’ FPKM> 3 in this tissue), and tongue flower tissue (1 gene, *CnMYB206*’ FPKM = 0.09 in this tissue) (Table S3). Of these 183 *R2R3-MYB* genes, transcript levels of 46 (*CnMYB219*, *CnMYB222*, *CnMYB152,* et al.*,)* and 57 (*CnMYB240*, *CnMYB222*, *CnMYB152,* et al.*,)* genes were higher in the flower bud than in the tubular flower and tongue flower, respectively. However, transcript levels of 31 (*CnMYB127*, *CnMYB308*, *CnMYB224,* et al.*,)* and 14 genes (*CnMYB127*, *CnMYB219*, *CnMYB21,* et al.*,)* were lower in the flower bud than in the tubular flower and tongue flower (Table S3).

Regarding the remaining *MYB* genes (*1R-MYB, 3R-MYB,4R-MYB,* and *atypical MYBs*), tissues from the root, stem, leaf, flower bud, tubular flower, and tongue flower all showed detectable transcript levels for 64 (*CnMYB15*, *CnMYB256*, *CnMYB151*, *CnMYB45*, *CnMYB217*, *CnMYB265*, *CnMYB26*, *CnMYB223*, *CnMYB61*, *CnMYB46*, and *CnMYB247)* genes were highly expressed in the root tissue (FPKM> 20), 76 (26 genes such as *CnMYB15*, *CnMYB181*, and *CnMYB126* were highly expressed in the stem tissue (FPKM> 20)), 71 (20 genes such as *CnMYB277*, *CnMYB245*, and *CnMYB126* were highly expressed in the leaf tissue (FPKM> 20)), 85 (17 genes, *CnMYB15*, *CnMYB45*, and *CnMYB61,* et al.*,* (FPKM> 20)), 70 (15 genes, *CnMYB126*, *CnMYB245*, and *CnMYB181,* et al.*,* (FPKM> 20)), and 68 (15 genes, *CnMYB126*, *CnMYB245*, and *CnMYB256,* et al.*,* (FPKM> 20)) out of the 141 genes, respectively (FPKM> 1). These *MYB* genes were expressed most in flower bud tissue and least in root tissue (Fig. S4, Table S4). An expression heatmap of other *MYB* genes was generated (Fig. S4). These *CnMYB* genes expression results showed that 71 genes (FPKM> 0) exhibited expression and 22 genes (FPKM = 0) were not expressed in any tissue (Table S4).

A total of 82 *CnMYB* genes (FPKM> 0) were expressed in all three flower tissues (Table S4). Among them, *CnMYB15*, *CnMYB45*, *CnMYB61*, *CnMYB256*, *CnMYB217*, *CnMYB151*, *CnMYB265*, *CnMYB126*, *CnMYB181*, and *CnMYB26* genes were highly expressed in the three tissues (FPKM> 20) (Table S4). We also identified genes that were specifically expressed in the bud (11 genes, *CnMYB40* and *CnMYB89* genes were highly expressed in this tissue (FPKM> 3)), tubular flower (7 genes, none of the genes were highly expressed in this tissue), and tongue flower tissue (1 gene, it’s FPKM = 0.093) (Table S4). Of these 141 *CnMYB* genes, transcript levels of 26 and 27 genes (such as *CnMYB15*, *CnMYB45*, and *CnMYB61,* et al.*,)* were higher in the flower bud than in the tubular flower and tongue flower. However, transcript levels of 20 (such as *CnMYB126*, *CnMYB245*, and *CnMYB181,* et al.*,)* and 15 genes (*CnMYB126*, *CnMYB245*, and *CnMYB256,* et al.*,)* were lower in the flower bud than in the tubular flower and tongue flower (Table S4).

**Fig. 5:**
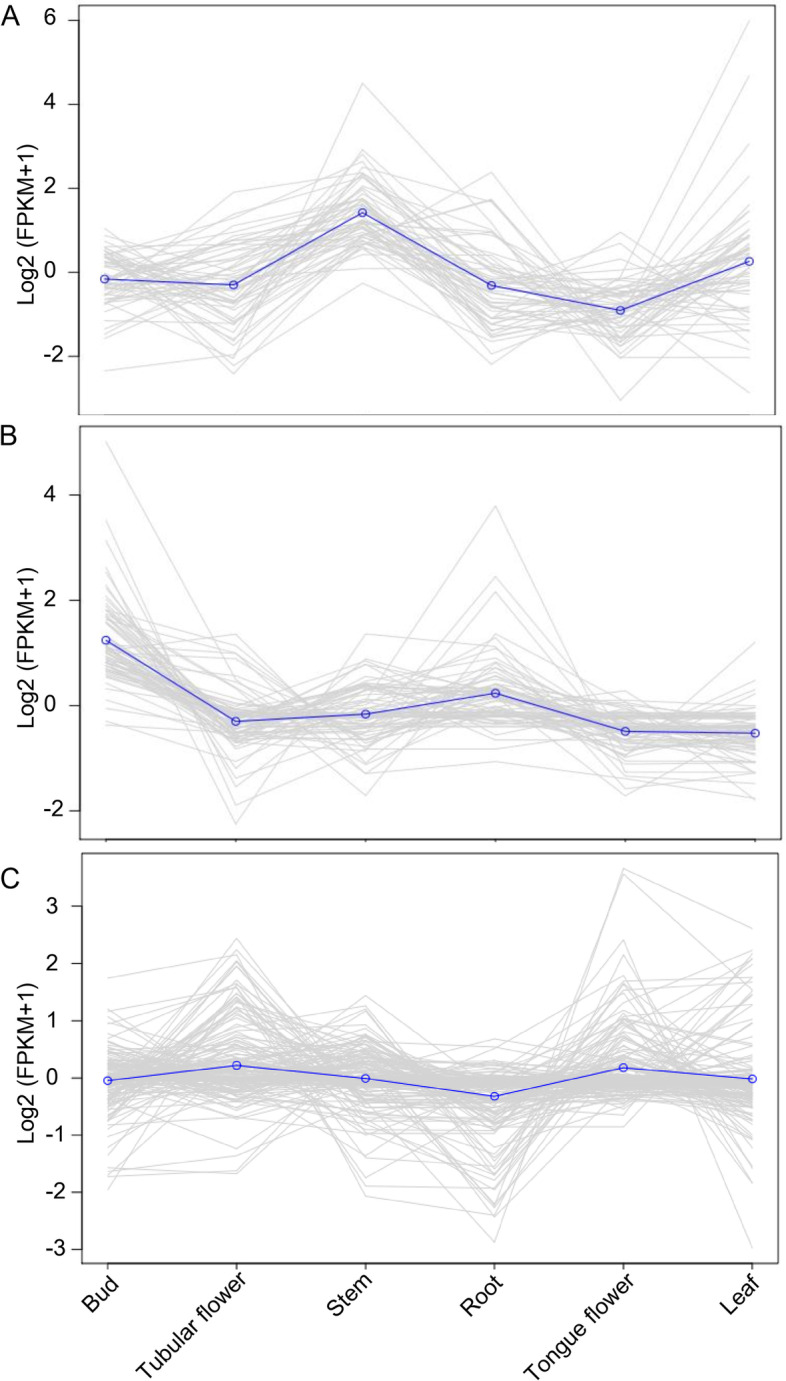
The trend analysis of 324 *CnMYB* genes expression (3 trends). **A** First trend. **B** Second trend. **C** Third trend

The expression type of all *CnMYBs* could be divided into three major patterns (Fig. 5, Table S5, S6, S7). The first trend (56 genes) had the highest expression in the stem (the FPKM values of *CnMYB28*, *CnMYB139*, *CnMYB60*, *CnMYB90*, *CnMYB247*, *CnMYB76*, *CnMYB180*, *CnMYB38*, *CnMYB10*, *CnMYB207*, *CnMYB314*, and *CnMYB153* genes were greater than 2) and lowest in tongue flower tissues (the FPKM values of *CnMYB28*, *CnMYB314*, and *CnMYB76* genes were less than − 2) (Fig. 5A, Table S5). The second trend (72 genes, *CnMYB222*, *CnMYB240*, *CnMYB322*, *CnMYB63*, *CnMYB282*, *CnMYB167*, *CnMYB111*, *CnMYB321*, and *CnMYB212* genes’ FPKM> 2) had the highest expression in the bud (Fig. 5B, Table S6). The members of the third trend (196 genes) had approximately similar gene expression patterns in all tissues (Fig. 5C, Table S7).

### Identification and expression analysis of *CnMYBs* in response to drought and salt stresses

**Fig. 6:**
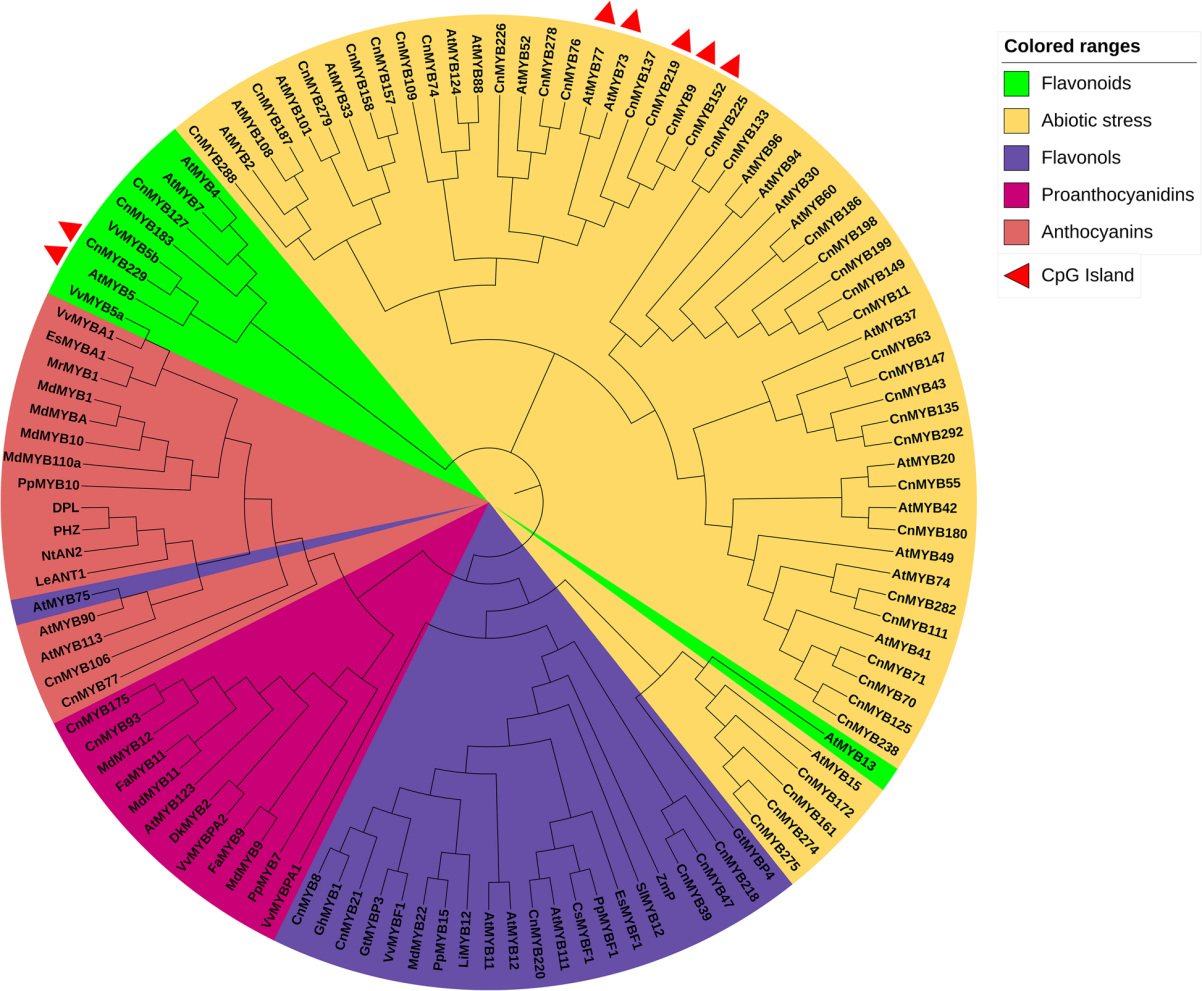
Analysis of *Chrysanthemum nankingense* phylogeny of MYBs in the Flavonoids, Abiotic stress, Flavonols, Proanthocyanidins, and Anthocyanins clades. All clades are represented by different colors and 4 *CnMYBs* and 3 *AtMYBs* genes containing CpG islands are marked with a red triangle

To identify additional CnMYBs participating in the regulation of drought and salt stress in Chrysanthemum, a phylogenetic tree that includes 51 CnMYB members (candidate genes can respond to stress resistance) and 66 functional MYBs regulating flavonoids, flavanols, proanthocyanidins, anthocyanins biosynthesis, and response to stress resistance from other species was generated (Fig. 6). According to Fig. 6, three CnMYBs were selected as candidates, potentially regulating the biosynthesis of flavonoids, i.e., CnMYB127, CnMYB183, and CnMYB229, six for flavonols, i.e., CnMYB8, CnMYB21, CnMYB220, CnMYB39, CnMYB47, and CnMYB218, two for proanthocyanidins, i.e., CnMYB175 and CnMYB93, two for anthocyanins, i.e., CnMYB106 and CnMYB77, and 38 CnMYBs may be involved in abiotic stress responses in *C. nankingense*. CpG islands were detected in four members (Fig. 6).

**Fig. 7:**
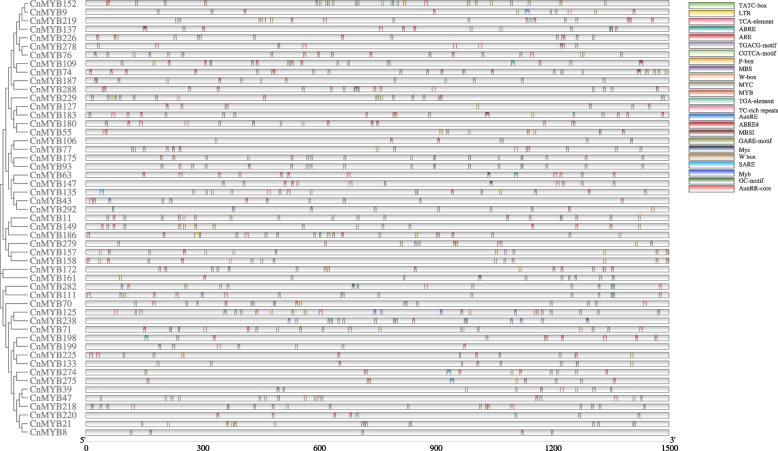
Predicted cis-acting elements related with stress response and hormones in 51 stress-resistant candidate *CnMYB* genes’ promoters. Promoter sequences (− 1500 bp) of *CnMYBs* were analyzed using PlantCARE. Different shapes and colors represent different elements and annotations of cis- acting elements are listed in Supplementary Table S9

To verify whether the candidate TFs had the ability to regulate abiotic stress responses in *C. nankingense*, the 1.5 kb sequence upstream of their start codons (ATG) of 51 selected *CnMYB* genes were submitted to the PlantCare online tool and searched for cis-acting elements. A diverse range of cis-acting elements was detected. Despite analyzing a huge number of cis-acting elements, we only focused on stress response elements according to our research objectives (Fig. 7, Table S8). These included anaerobic induction (ARE), drought stress (MYB, MYC, W-box, and MBS), salinity stress (TC-rich repeats), low temperature-responsive (LTR), abscisic acid-responsive (ABRE), and salicylic acid-responsive (TCA) cis-acting elements. Most of the *CnMYB* promoters contained ARE, ABRE, MYB, and MYC cis-acting elements. The proportion of ABRE was the largest amongst the cis-acting elements. Promoters of 51 stress-responsive candidate genes contain more or less stress response elements (Fig. 7, Table S8).

**Fig. 8:**
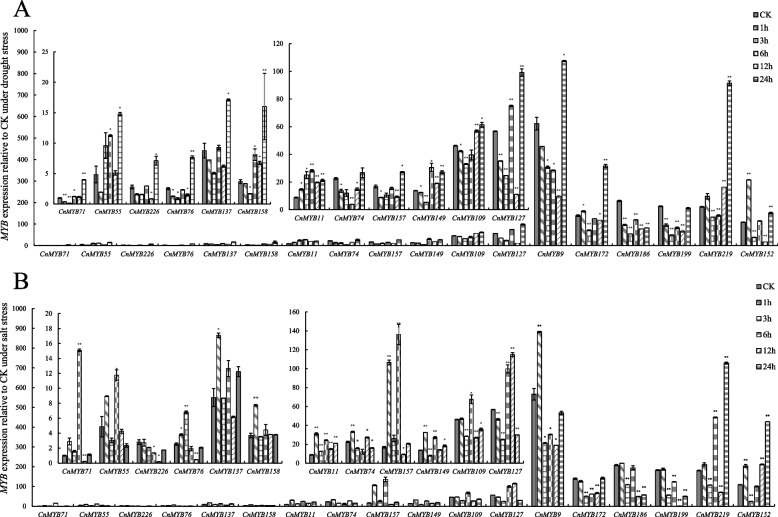
The expression of 19 *CnMYBs* was induced by abiotic stresses. The eight leaves seedlings were treated with 15% PEG6000 (**A**), 150 mM NaCl (**B**) for qRT-PCR. Samples were taken at 0, 1, 3, 6, 12, and 24 h after the stress treatment. *CnActin* gene was used as internal reference. The error bar is standard deviation (*n* = 3; *P < 0.05; **P < 0.01; Student’s t test)

To investigate the function of these *CnMYB* genes in *C. nankingense*, the expression patterns of 18 candidate genes that contained ARE and ABRE two stress response elements were analyzed under drought and salt stresses. The expression levels of the 18 selected *CnMYBs* were detected by qRT-PCR at 0 h, 1 h, 3 h, 6 h, 12 h, and 24 h after these treatments. All genes of the 18 *CnMYBs* showed fluctuating changes in their transcript levels after the drought stress treatments (Fig. 8A). *CnMYB9*, *CnMYB76*, *CnMYB109*, *CnMYB137*, *CnMYB157*, *CnMYB186*, and *CnMYB199* were significantly down-regulated by drought stress, however, except *CnMYB186*, others genes expression increased to the highest level at 24 h after treatment. The expression of *CnMYB172* and *CnMYB11* was significantly up-regulated after drought treatment. The expression of *CnMYB55*, *CnMYB149*, *CnMYB158*, and *CnMYB127* was lower than CK at the initial stage of drought treatment but gradually increased with increasing stress time and reached a peak at a later stage, suggesting that these *CnMYBs* may be involved in responses to drought stress.

All 18 *CnMYBs* showed fluctuating changes in their transcript levels after the salt stress treatments (Fig. 8B). *CnMYB199*, *CnMYB186*, and *CnMYB172* were significantly down-regulated by salt stress. The expression of *CnMYB11* and *CnMYB137* was significantly up-regulated after salt treatment and reached its peak at 1 h. The expression of *CnMYB9*, *CnMYB158*, and *CnMYB76* was higher than CK at the initial stage of salt treatment but gradually decreased with increasing stress time. The expression of *CnMYB219*, *CnMYB55*, *CnMYB149*, and *CnMYB157* increased first, then decreased, and then increased with increasing stress time, suggesting that these *CnMYBs* may be involved in responses to salt stresses.

Based on the levels of expression under drought and salt stress, 18 genes were sorted into three categories (when the treatment time was zero, the expression of the *CnMYB71* gene was set to one, as a control), among them, *CnMYB9*, *CnMYB172*, *CnMYB199*, *CnMYB219*, *CnMYB186*, and *CnMYB152* genes were highly expressed, *CnMYB11*, *CnMYB74*, *CnMYB157*, *CnMYB149*, *CnMYB109*, and *CnMYB127* genes were moderate, *CnMYB71*, *CnMYB55*, *CnMYB226*, *CnMYB76*, *CnMYB137*, and *CnMYB158* genes have relatively low expression levels (Fig. 8). All 18 genes were regulated by drought and salt stress, but the genes with moderate and high expression levels will be the focus of future research.

*CnMYB9*, *CnMYB152*, and *CnMYB219* have CpG islands in the gene body (Fig. 6), and the methylation status detected by MSP was analyzed through gel electrophoresis. The expression of *CnMYB9*, *CnMYB152*, and *CnMYB219* was regulated under drought and salt stresses. Total genomic DNA of *C. nankingense* was extracted from the leaf of the treated seedlings with drought and salt stresses at the duration of was 0, 1, and 6 h. Gene expression varied greatly compared with CK in these tissues. The MSP results showed the presence of methylation in *CnMYB9* and *CnMYB152* in leave tissues at different stress times, but the *CnMYB219* gene is demethylated at three stress times. So the methylation status of the three genes’ CpG islands did not change under drought and salt stress treatments in three stress times (Fig. S5).

## Discussion

In all eukaryotes, the MYB gene family participates in a variety of biological activities, such as metabolism, plant development, and responses to biotic and abiotic stimuli [1]. Numerous species, including model plants [1, 3, 24], significant crops [2, 4, 12], and other plants have been studied in relation to the MYB family. Different plants had varying numbers of MYB family members, including 198 in *Arabidopsis* [1], 379 in soybeans [35], 129 in the Chines pear [36], 170 in grape [37], and 174 in Chinese bayberry [5]. Here, 324 *CnMYB* genes from the *C. nankingense* genome were discovered using bioinformatics research (Fig. 1, Table S1). As a result, there was no correlation between the number of *MYB* genes and genome size. Based on phylogenetic research, the *R2R3-MYB* gene family from *C. nankingense* was divided into 32 subgroups, most of which had conserved motif compositions and exon-intron organizations except for the C26 and C28 subgroups, which showed the need for species to adapt to their particular environments (Figs. 2, 3). This outcome is somewhat consistent with the earlier reports [38]. Other MYBs from *C. nankingense* were classified into 16 subgroups based on phylogenetic analysis, with a certain degree of divergent conserved motif compositions and exon-intron organizations (Fig. S2).

Based on transcriptome data, we analyzed the expression patterns of 324 *CnMYB* genes in six tissues and divided them into 3 trends according to different expression patterns (Fig. 5). The expression levels of 60% *CnMYBs* (196 genes) are similar across all tissues (Third trend) (Fig. 5C). Second trend has 72 genes (22%) and highly expressed in the bud tissues (Fig. 5B). 20% of *CnMYB* (50 genes) genes are highly expressed in the stem (Fig. 5A). This result indicated that CnMYBs might perform diversified functions during the *C. nankingense* lifespan.

Lodging is one of the most important agronomic traits that play an essential role in the quality and yield of crops [39, 40]. Cellulose and lignin contents of plant stems can affect the resistance of plants against lodging [39, 41]. A previous study reported that AtMYB46 and AtMYB83 are considered secondary main switches for controlling secondary cell wall biosynthesis [42] AtMYB46 and AtMYB83 can induce the expression of cellulose and lignin synthesis genes to promote the thickening of the secondary wall and their downstream MYB TFs (AtMYB58, AtMYB63, and AtMYB85) also play an important role in the regulation of secondary wall biosynthesis [43–45]. Moreover, there are also some MYB TFs, such as AtMYB52, AtMYB54, AtMYB69, and AtMYB103 may also be involved in the regulation of secondary cell walls [44]. Except, for positively regulating second cell wall synthesis genes of MYB transcription, AtMYB7, AtMYB4, and AtMYB32 may be negative regulators of lignin synthesis [46–49]. The combined transcriptomic data and phylogenetic tree analysis showed that CnMYB278, CnMYB226, CnMYB180, CnMYB76, CnMYB127, and CnMYB219 may be involved in the regulation of cellulose and lignin synthesis (Fig. 2, Table S7). Among them, the *CnMYB278* was specifically expressed in the tubular flower tissue, and the *CnMYB127* and *CnMYB219* genes were highly expressed in all tissues. While *CnMYB226*, *CnMYB180*, and *CnMYB76* genes were highly expressed in the stem and slightly expressed in other tissues. These results suggest that *CnMYB226*, *CnMYB180*, and *CnMYB76* genes should be considered important candidate genes involved in the regulation of cellulose and lignin synthesis in stem tissue (Table S7).

MYB transcription factors family is one of the largest transcription factor families in plants. Some MYB TFs have effects on flowering time, anther, and pollen development [15, 16], while other MYB proteins have been found to participate in secondary metabolism processes, such as the biosynthesis of proanthocyanidins, anthocyanins, flavonols, and flavonoids in plants. These metabolites directly affect the color of flowers and fruits, so some MYB TFs are involved in flower color development [8, 50–57]. In this study, we identified a number of *CnMYB* genes that had higher expression levels in flower bud tissue are higher than in tubular flower and tongue flower tissues (Table S7). These results indicate that most CnMYB were involved in the flower bud development. The combined genes’ higher expression in flower tissues of transcriptomic data and phylogenetic tree analysis showed that CnMYB279, CnMYB288, CnMYB157, CnMYB9, CnMYB152, and CnMYB219 TFs may be involved in the regulation of flowering time, anther, and pollen development (Fig. S2). CnMYB175, CnMYB93, CnMYB229, CnMYB8, CnMYB106, CnMYB39, CnMYB21, CnMYB127, CnMYB47, and CnMYB183 TFs may participate in the regulation of flower color (Fig.6).

Numerous prior investigations [1, 11, 17, 58] have documented the crucial involvement of MYB TFs in the *Arabidopsis* stress response, particularly in response to abiotic stressors. Secondary Phenylpropanoid metabolites have been widely documented as defense metabolites for their role as antioxidants in protecting plants from abiotic stressors by scavenging reactive oxygen species [59]. In this study, we integrated stress response and phenylpropanoid secondary metabolite proteins with CnMYB proteins to build a phylogenetic tree (Fig. 6). We selected 51 CnMYB proteins that may be involved in abiotic stress response in *C. nankingense* based on their similarities.

Analysis of cis-elements on gene promoters is very important because it allows predicting the functional role of the genes [60, 61]. A large number of cis-acting regulatory elements were obtained from these 51 *CnMYB* genes promoter (Fig. 7), including MYC recognition site, WRKY recognition site, MYB recognition site, MYB binding site involved in drought-inducibility (MBS) [62], MYB binding site involved in the flavonoid biosynthetic genes regulation (MBSI), low-temperature responsiveness (LTR) [63], abscisic acid responsiveness (ABRE) [61, 64, 65], anaerobic induction site (ARE), MeJA responsiveness (TGACG-motif and CGTCA-motif), defense and stress responsiveness (TC-rich repeats) that are known to involve in various plant development and stress response functioning [66, 67]. The combined phylogenetic tree and stress-related cis-elements analysis would help us in selecting stress response candidate genes.

To further validate the stress-related candidate genes, we determined the expression patterns of 18 genes from 51 candidate *CnMYB* genes under drought and salt stresses by analyzing qRT-PCR. Eighteen *CnMYB* genes expressed differently at different time points, these patterns are similar to *AtMYB* genes in Arabidopsis under drought and salt stresses (Fig. 6). We focused on moderately and highly expressed genes. Among them, CnMYB127 clustered with Arabidopsis AtMYB4 and AtMYB7, indicating that CnMYB127 can negatively regulate abiotic stress [19, 26, 68]. CnMYB11, CnMYB186, CnMYB199, and CnMYB149, as AtMYB60 homologs; AtMYB88, and AtMYB124, as CnMYB109 homolog; CnMYB219, CnMYB9, and CnMYB152, as AtMYB77 homologs; AtMYB13, and AtMYB15, as CnMYB172 homologs; AtMYB33 as CnMYB157 homolog, can positively regulate some abiotic stress [29, 30, 69–73]. These 18 genes were randomly selected from 51 stress-resistant candidate genes and the stress-resistant functions of the other 33 genes need to be further verified, but the stress resistance of these 18 genes has demonstrated the accuracy of our predicted stress-resistant candidate genes.

There are many reports on MYB TFs’ regulation of abiotic stress in the plant [17, 30, 69, 70, 73], but its link with epigenetic control is rarely reported [30, 31, 74]. As CG or CpG is the most abundant source of DNA methylation, methylation-sensitive amplification polymorphism and methylation-specific PCR (MSP) are two methods that have been applied to methylated genes [75, 76]. Here we have identified 44 genes that may alter the epigenetic makeup of chrysanthemum by having high CpG content in the promoter and gene body regions (Fig. 1, Table S2). Four of the 51 stress response candidate genes had CpG islands, among them, *CnMYB219*, *CnMYB9*, and *CnMYB152* were identified on the basis of methylation-specific PCR (MSP) results (Fig. 6, Fig. S5). These three genes can regulate gene expression and may play a role in drought and salt stresses, as their expression significantly changed under stress conditions (Fig. 8). We only examined three brief treatment intervals to identify methylation; it would be preferable to investigate the methylation status over a wider range of treatment times in the future. Our future research will focus on the regulation of flower development and drought and salt stress functional candidate genes.

## Conclusions

In this study, 324 *MYB* genes were identified in the genome of *C. nankingense*. A comprehensive bioinformatics analyses were performed to investigate phylogenetic relationships, conserved motifs, gene structure, and promoter analysis. The present study investigated the expression patterns of these genes in different tissues by analyzing RNA-Seq data. *CnMYB226*, *CnMYB180*, and *CnMYB76* genes were selected to further explore the regulation of cellulose and lignin synthesis in stem tissue. The regulation of flowering time, anthers development, pollen formation, and flower colors may be mediated by 16 *MYB* genes. We found 51 candidate genes by bioinformatics analysis that may be involved in stress resistance, and we also confirmed 18 of these candidate genes through qRT-PCR. According to gene expression levels under stress conditions, six *CnMYB* genes were identified as key stress-responsive genes. The information provided by these results may be helpful for further functional analysis of *CnMYB* gene to elucidate its development and abiotic stress mechanism in *C. nankingense*.

## Methods

### Plant growth conditions and treatments

The wild *C. nankingense* plants (the deposit number is NEAU0006698) were obtained from the Plant Germplasm Resources and Genetic Engineering, Henan University, Kaifeng, Henan Province, China. The plants were planted in the growth room of the Plant Germplasm Resources and Genetic Engineering, Henan University, and were maintained under the following conditions: 25 °C, with a 16 h light /8 h- dark cycle. The materials were propagated by cuttings and grown in pots. Seedlings with more than eight leaves were treated with two stress conditions: 15% PEG6000, and 150 mM NaCl. The duration of treated Seedlings with two stress conditions was 0, 1, 3, 6, 12, and 24 h, and then used for detection of gene transcript levels. A sample with 0 h duration served as the control. Each experiment was repeated three times.

### Database searches, sequence retrieval, and gene identification

The *C. nankingense* genome and protein sequences were downloaded from the *Chrysanthemum* genome database (http://www.amwayabrc.com). The Hidden Markov Model (HMM) profile of the MYB DNA binding domain (PF00249) was downloaded from the Pfam protein family database (http://pfam.xfam.org/), then used it as a query (*p* < 0.001) for the identification of all putative *CnMYB* genes [77]. The SMART database (http://smart.embl-heidelberg.de/), the NCBI Conserved Domain database (http://www.ncbi.nlm.nih.gov/Stucture/cdd/wrpsb.cgi), and the Pfam database (http://pfam.janelia.org/) program were exploited to test for the presence of the MYB domain. The number of amino acids, open reading frame (ORF) length, molecular weight (MW) and isoelectric point (pI) of CnMYBs were obtained through the ExPASy (http://www.expasy.ch/tools/pi_tool.html) [78]. *A. thaliana* genome sequences were obtained from TAIR (http://www.Arabidopsis.org/) [79].

### Phylogenetic analyses of CnMYBs

The protein sequences of CnMYB proteins were aligned by the ClustalW program and adjusted manually, the phylogenetic tree was constructed by the neighbor-joining method of MEGA 7.0 with 1000 bootstrap replicates [80]. All subsequent phylogenetic trees were constructed by the same method as above.

### Gene structure and protein motifs analysis of the *CnMYB* gene family

The exon, intron and UTR of the *CnMYB* genes were determined by the Gene Structure Display Sever (GSDS 2.0) (http://gsds.cbi.pku.edu.cn/index.php) using the genomic sequences and coding regions, including intron numbers [81]. The multiple expectations for motif elicitation (MEME) online program (http://meme-suite.org) was used for the identification of conserved motifs in the CnMYB protein sequences, with the following parameters: maximum number of motifs 10, minimum motif width 6, and maximum motif width 200 [82]. The MEME results were also visualized by TBtools software [83].

### CpG island analysis of the *CnMYB* gene family

“Methyl Primer Express V1.0” software (Methyl Primer Express Software v 1.0 Quick Reference Card (thermofisher.com)) was used for the CpG islands analysis of a 1500 bp upstream sequence and DNA (coding protein region). The conditions set for analysis are as follows: the maximum length of the CpG island was 2000 bp, the minimum length was 200 bp; the (C + G)/total bases ratio was 0.5; the criterion of CpG Observed/CpG Expected” was 0.6. The sequences were submitted to software to identify CpG islands or rich regions [84].

### Prediction of cis-acting elements in promoter regions

PlantCARE (http://bioinformatics.psb.ugent.be/webtools/plantcare/html/) was used for the cis-acting analysis of a 1500 bp upstream sequence of the identified candidate *CnMYB* genes in the abiotic stress response. TBtools software was used to display the number of identified elements.

### RNA-seq based expression analysis of *MYB* genes in six different tissues

We downloaded raw RNA-Seq data of leaf, stem, root, floral bud, disc florets, and ray florets from the chrysanthemum genome database (http://www.amwayabrc.com) [23]. A value for fragments per kilobase of transcript per million fragments mapped (FPKM) was calculated for each gene, and the log2 (Fold Change) transformed values for each *CnMYB* family gene were used to generate a heatmap. A heatmap was generated and visualized using the TBTOOLS software [83], the color scale shown represents log2 transformed FPKM values. R software (https://www.r-project.org) was used for the clustering analysis.

### Quantitative real-time polymerase chain reaction (qRT-PCR) analysis

Total RNA was extracted from leaves using the RNAprep Pure Plant Plus Kit (spin column) (Takara, Dalian, China) based on specifications. Approximately 1 μg total RNA was reverse-transcribed into cDNA using the “StarScript ǁ First-stand cDNA Synthesis Mix with gDNA Remover”. The qRT-PCR was performed on using a Roche LightCycler 480II detection system, using Hiscript II one-step RT-PCR SYBR Green kit (Vazyme, Nanjing, China). The primers used in this study are listed in Supplementary Table 1. Each sample was conducted in three biological replications. *CnActin* (KF305683) was used as the reference gene. The relative expression level of each gene was calculated as 2^-ΔΔCT^ equation [85].

### Methylation-specific polymerase chain reaction (MSP)

Total DNA was extracted from leaves using “Plant Genomic DNA Kit” (TOLOBIO) based on specifications. The integrity and concentration of the DNA was verified by 1% agarose gel electrophoresis and NanoDrop™ 2000/2000c (ThermoFisher Scientific, USA). Total DNA was treated with sodium bisulfites using “DNA Bisulfite Conversion Kit” (Tiangen) based on specifications. Sodium bisulfites treated DNA was used as the template for methylation-specific PCR. The methylated and unmethylated primers of CpG islands were designed using “Methyl Primer Express Version 1.0” software (Table S9). The PCR product length was between 100 and 175 bp. Three biological replicates and 35 cycles for each reaction were performed [84].

## Supplementary Information

**Additional file 1: Table S1.** The isoelectric point, protein length, molecular weight, Exon numbers, scaffold No, and MYB-domain type of the members of CnMYB gene family. CnMYB genes were named according to the order of scaffold No. **Table S2.** Details of each of the 41 genes with CpG island: the 324 CnMYB genes were analyzed for CG-rich regions using “Methyl Primer Express V1.0” software. **Table S3.** List of R2R3-CnMYB genes expression in different tissues. **Table S4.** List of CnMYB (1R-MYB, 3R-MYB,4R-MYB, and atypical MYBs) genes expression in different tissues. **Table S5.** List of First trend’ CnMYB genes expression in different tissues. **Table S6.** List of Second trend’ CnMYB genes expression in different tissues. **Table S7.** List of Third trend’ CnMYB genes expression in different tissues. **Table S8.** List of main motif numbers in 51 stress-resistant candidate genes’ promoters. **Table S9.** List of the primers used for qRT-PCR and MSP alaysis in this study.

**Additional file 2.**

**Additional file 3.**

**Additional file 4.**

**Additional file 5.**

**Additional file 6.**

## Data Availability

The *C. nankingense* genome and protein sequences were downloaded from the *Chrysanthemum* genome database (http://www.amwayabrc.com). Other data have been submitted as supplementary materials. All data analyzed or generated of this study are available from the first or corresponding author on reasonable request.
