# Supplementary figures and images for: Genome-wide characterization and expression analysis of MYB transcription factors in Chrysanthemum nankingense
Source: BMC Plant Biol. 2023 Mar 14;23:140. doi: 10.1186/s12870-023-04137-7 (PMC10012607; doi:10.1186/s12870-023-04137-7)

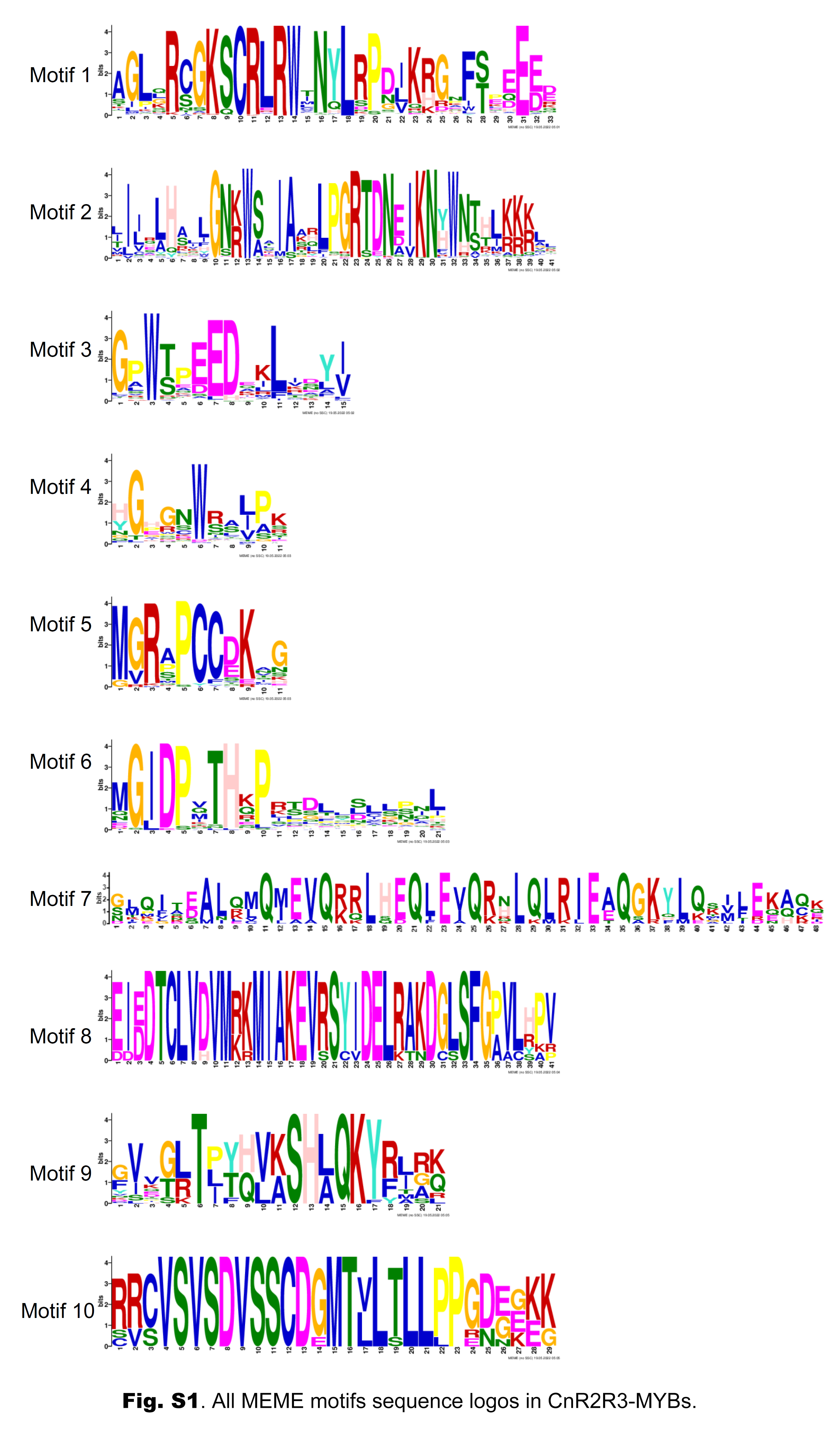

Supplement: Supplementary file 2 — Additional file 2. [file 12870_2023_4137_MOESM2_ESM.tif]

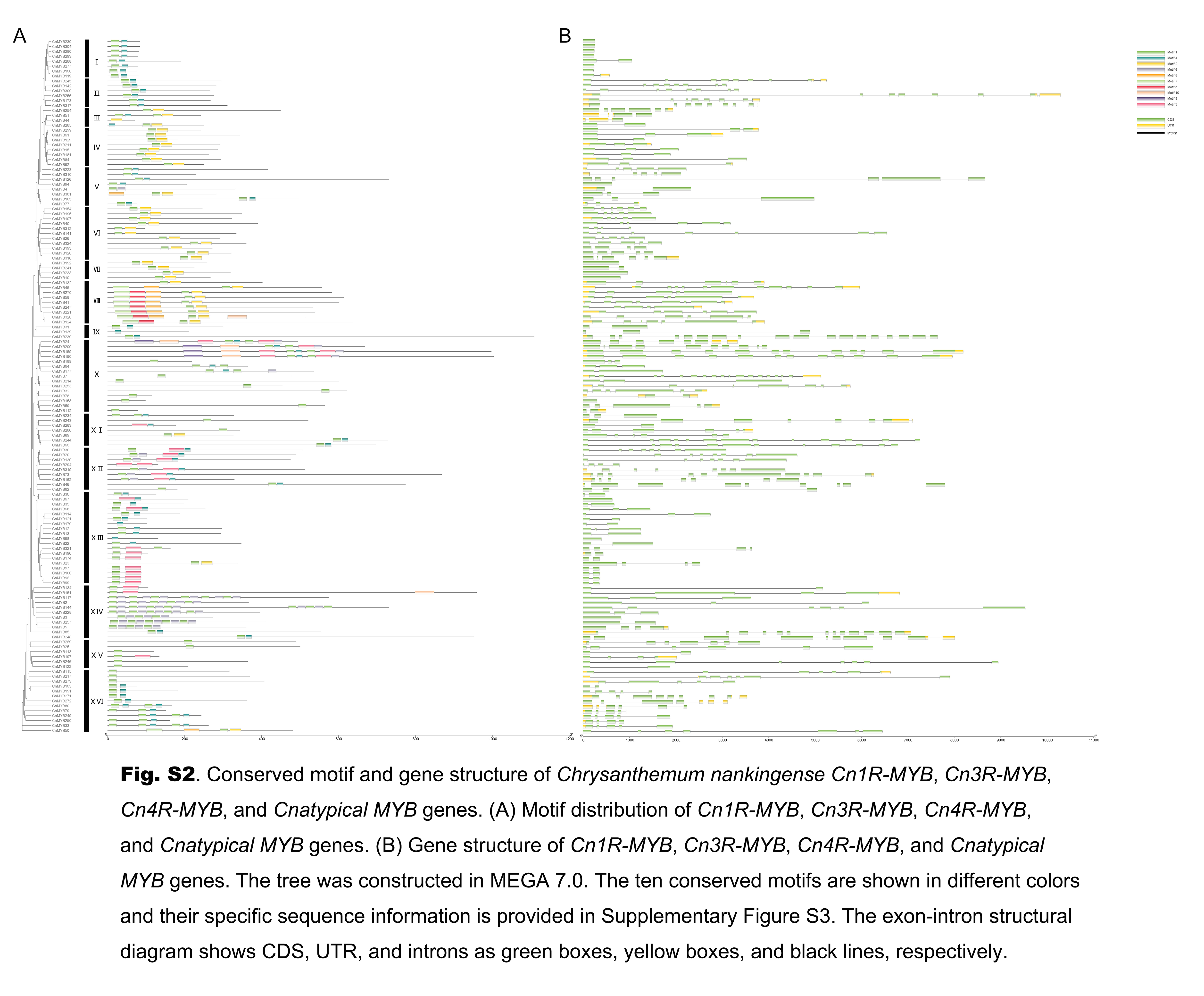

Supplement: Supplementary file 3 — Additional file 3. [file 12870_2023_4137_MOESM3_ESM.tif]

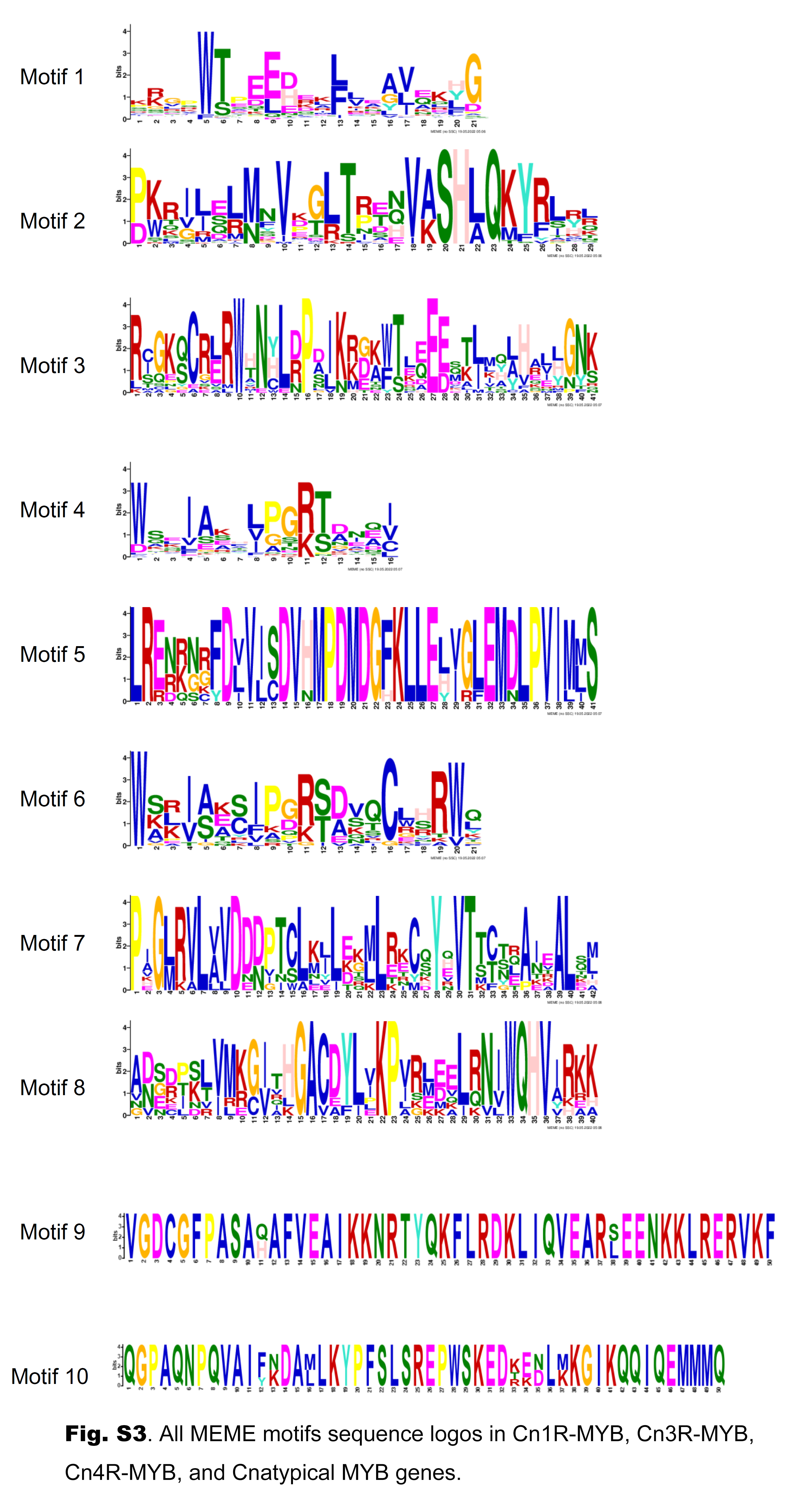

Supplement: Supplementary file 4 — Additional file 4. [file 12870_2023_4137_MOESM4_ESM.tif]

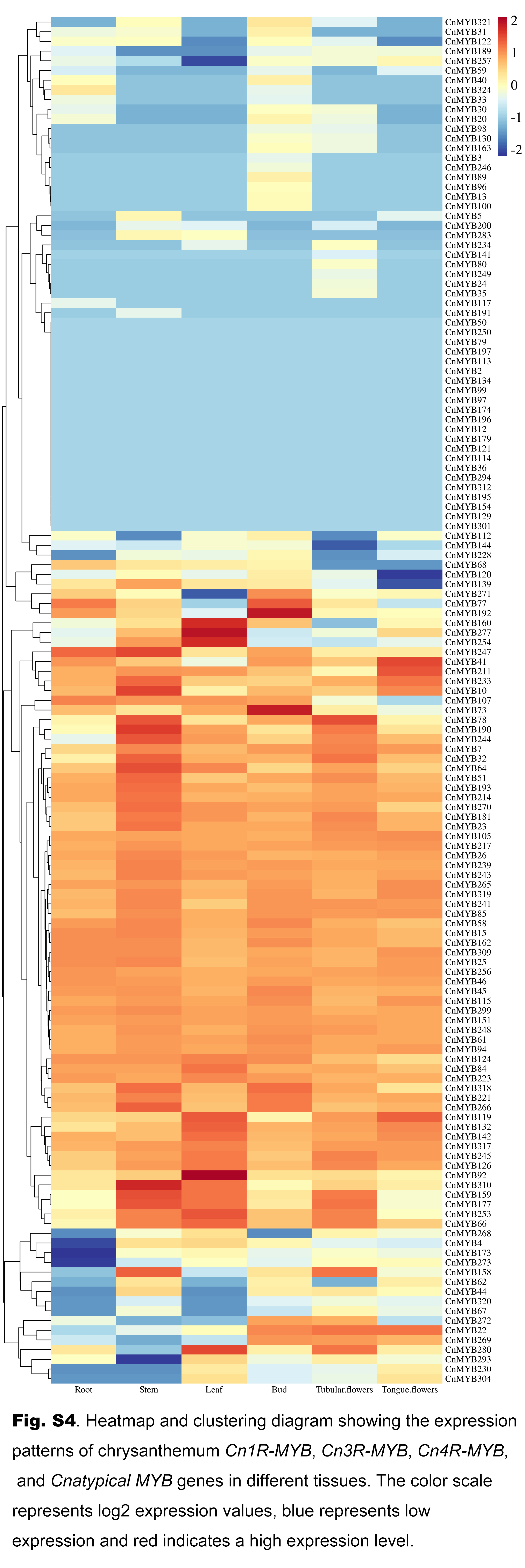

Supplement: Supplementary file 5 — Additional file 5. [file 12870_2023_4137_MOESM5_ESM.tif]
